# Using smart transportation assets to hedge fossil energy markets: Evidence from quantile-based VAR approach

**DOI:** 10.1371/journal.pone.0317748

**Published:** 2025-05-09

**Authors:** Md. Bokhtiar Hasan, Md. Naiem Hossain, Hosneara Khatun, Gazi Salah Uddin, Mehmet Huseyin Bilgin, Shu Tian

**Affiliations:** 1 Department of Finance and Banking, Islamic University, Kushtia, Bangladesh; 2 Department of Economics and Finance, University of New Orleans, United States of America; 3 Department of Tourism and Hospitality Management, Islamic University, Kushtia, Bangladesh; 4 Department of Management and Engineering, Linköping University, Linköping, Sweden; 5 Faculty of Political Sciences, International Relations, Istanbul Medeniyet University, Istanbul, Turkey; 6 Economic Research and Development Impact Department, Asian Development Bank, Philippines; Bucharest University of Economic Studies: Academia de Studii Economice din Bucuresti, ROMANIA

## Abstract

This study explores the time-varying correlations and quantile spillover connectedness to identify the hedging potential of smart transportation assets for energy markets. The study finds that amid crises like COVID-19 and the Russia-Ukraine conflict, smart transportation indices demonstrate strong safe-haven characteristics against volatility in equity commodity energy and electricity transmission and distribution infrastructure indices. Additionally, the smart transportation, electric vehicle, and drone indices offer limited hedging benefits and safe-haven attributes for carbon emission allowance and natural gas. Furthermore, smart transportation assets are the major spillover transmitters to fossil energy assets across all quantiles. These outcomes hold substantial implications for environmental advocates, investors, and policymakers.

## 1. Introduction

Climate change is causing rising concern in the world today since the globe is already witnessing the disastrous repercussions of climate change in the form of major natural catastrophes such as heatwaves, droughts and wildfires, floods, and tsunamis, among other things [1,2]. Meanwhile, CO_2_ emissions are widely regarded as the primary cause of climate change. However, along with rapid urbanization [3], the transportation industry is one of the major emitters, accounting for around 21% of worldwide CO_2_ emissions in 2016 [4]. These emissions are primarily caused by using fossil fuels in road, rail, aviation, and marine transportation [5,6, 7]]. Transport demand is rapidly increasing as the world population grows. If this trend continues, it may have significant ramifications for human health and energy security, as most vehicles still rely heavily on fossil fuels [8].

**Fig 1:**
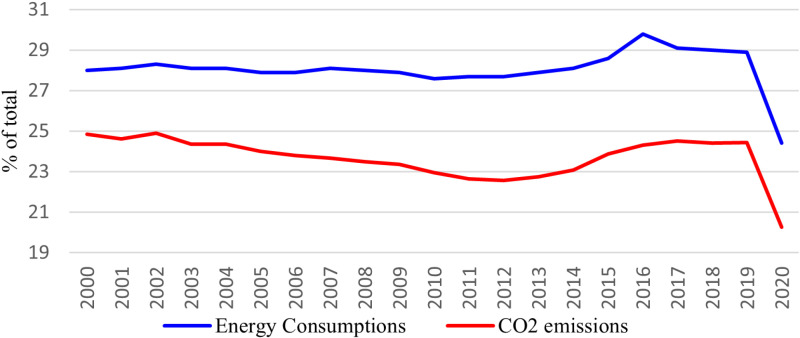
Share of total final energy use and CO_2_ emissions by the global transportation sector.

During the past two decades, the transportation industry consumed around 28% of the total fossil energy globally. It emitted approximately 23–24% of total CO_2_, with a significant drop in 2020 owing to the COVID-19 lockdown (see Fig 1). As a result, both energy consumption and CO_2_ emissions by the transportation sector are relatively high, garnering global attention to curb the transportation industry’s excessive reliance on fossil fuels. Reducing this sector’s emissions is projected to have a significant role in meeting the net-zero emission targets. Meanwhile, many countries have already taken steps to reduce fossil fuel use in transportation. According to the International Energy Agency (IEA), smart transportation—not based on fossil energy and combining internet, wireless technology, and electronic gadgets for more sustainable and efficient travel—is one of the best solutions for lowering the transportation sector’s heavy reliance on oil. Similarly, the Chinese Ministry of Transport has launched a project titled “The Plan of Smart Transportation to Make Travel More Convenient Action (2017-2020),” which lays out the roadmap for developing smart transportation in China (Zhao et al., 2022). Likewise, the United States is already on pace [9,10].

Global light vehicle sales have lately surged dramatically due to the enforcement of mandatory vehicle carbon emissions standards in several countries, such as the United States, China, and Japan, spanning more than a decade. The statistic shows that more than 70% of light vehicles are sold because of their emissions standards. However, this worldwide smart transportation demand trend is expected to triple by 2050, reducing the transportation sector’s reliance on fossil fuels to zero, albeit power consumption may climb [11,12]. Hence, it is speculated that there may be an opposite connection between smart transportation and fossil-based energy markets. More clearly, if investment in smart transportation markets increases due to transportation innovation, investments in fossil energy markets may suffer [13]. Thus, two opposite sectors may have volatility spillovers, which need to be explored empirically.

Recent global crises such as the Russia-OPEC price war, the COVID-19 lockdowns worldwide, and the ongoing Russia-Ukraine conflict have severely impacted global energy markets, particularly in natural gas and oil, causing unprecedented volatility [12,14–16]. These volatility shocks have heightened uncertainty among investors in the energy sector, diminishing the hedging effectiveness of traditional assets [12,14,16–21]. In such turbulent times, investors typically seek alternative assets with safe-haven qualities to safeguard their portfolios from financial losses.

In this vain, green ethical assets like green bonds, green equities, and clean equities have emerged as viable alternatives due to their potential to act as hedges or safe havens, especially during market downturns [22–26], and their positive environmental impact [27]. It is noted that green assets are ethical assets, and ethical investors typically do not switch from ethical to unethical assets during times of crisis, instead opting for buy-and-hold tactics. Furthermore, green bond investors historically included large long-term investors such as pension funds and insurance firms, and most green bond issuers are also large institutions with well-structured governance [28,29]. As a result, green assets are less vulnerable to crises and function as a safe haven. Accordingly, smart transportation is a green asset that saves energy and reduces CO_2_ emissions by improving energy efficiency [8,30]. Hence, it is speculated that this asset class may also provide hedging and safe-haven properties that require empirical verification, particularly against fossil energy market shocks.

However, the extant literature on the connectedness between smart transportation and fossil energy investment is very scant, with the majority focused on individual countries. For example, Zhao et al. [30] recently evaluated the influence of smart transportation on carbon emission reduction in China, demonstrating that smart transportation has a strong upward trend and a spatial spillover effect on carbon emissions. In contrast, [31] indicated that fossil-fuel reliance in the US transportation sector is still difficult to balance with renewable energy. Nevertheless, Karami and Kashef [32] proposed a parameter for future study in smart transportation planning, concentrating on diverse data sources, forecasting models and their features, and future commercial potential. Duan et al. [33] studied electric transportation systems and distributed energy resources in a smart city. The authors showed that optimizing electric vehicles may lower total energy costs while enhancing vehicle efficiency.

From the above literature discussions, it can be said that a few studies have empirically tested smart transportation to look into its influence on energy usage and CO_2_ emissions, with varied outcomes. Conversely, the other studies are primarily descriptive in nature. It seems that no study has examined the hedging properties of smart transportation assets yet, particularly for defending against shocks in fossil energy markets. In addition, no evidence of return volatility spillovers between them is found as well. Hence, a comprehensive study is desired in the literature to comprehend smart transportation’s hedging properties to safeguard investors from the current havoc in the global fossil energy markets.

Furthermore, global climate concerns and increasing environmental awareness are steadily driving the integration of sustainable investment concepts into traditional portfolios, including those involving fossil energy. Consequently, investors are progressively seeking opportunities that align with their environmental and social values, fostering the growth of eco-friendly and impact investment trends [34]. This shift reshapes the investment landscape, encouraging companies to adopt more socially responsible and environmentally sustainable practices.

In light of these above phenomena, our study investigates the dynamic quantile spillover effects between five smart transportation stock indices—Smart Transportation Index (SMTR), Autonomous Vehicles (AUVE), Electric Vehicles (ELVE), Advanced Transport Systems (ADTR), and Drones Index (DRON)—with five fossil energy stock indices—Equity Commodity Energy Index (COME), Carbon Emission Allowances (CEMA), Natural gas (NATG), Electricity Transmission and Distribution Infrastructure index (ELTR) and GSCI petroleum index (PTRL). As discussed above, smart transportation and fossil energy markets are opposite asset classes and are likely to be decoupled, signifying the hedging or safe-haven potential.

Utilizing dynamic conditional correlation (DCC) and a novel quantile vector autoregression (QVAR) spillover method, our research reveals that smart transportation returns and COME and PTRL returns exhibit significant volatility clustering, with both short-term and long-term spillover effects. During periods of stress, such as the COVID-19 pandemic and the Russia-Ukraine conflict, smart transportation indices demonstrate a robust safe-haven potential against spillover shocks in COME and ELTR. Additionally, smart transportation indices show effective hedging capabilities against ELTR over the entire study period. Furthermore, SMTR, ELVE, and DRON provide limited hedge and safe-haven opportunities against CEMA and NATG shocks. Our findings further suggest that smart transportation assets are the main sources of spillovers to energy assets across different quantiles. Conversely, COME and CEMA emerge as the primary recipients of these spillovers during extreme market conditions.

Our study enhances the extant smart transportation literature in several ways. First, our study is the maiden attempt to look at the hedging and safe-haven characteristics of smart transportation within the fossil fuel energy market context. We also investigate the spillover attributes of smart transportation to fossil energy markets. Second, our findings indicate that smart transportation assets offer a safe haven and hedging elements for fossil fuel energy markets. These findings may entice greater investments in the smart transportation sector from both environmentally concerned and traditional investors, indirectly reducing reliance on fossil fuel energy. Plummeting fossil fuel consumption may be a boon in terms of lowering CO_2_ emissions. Consequently, our findings suggest that smart transportation investors will have the opportunity to contribute to reducing environmental stress while simultaneously benefiting from the optimal investment opportunity. Lastly, unlike traditional methods focusing on overall time connectivity, our use of quantile connectedness allows for a nuanced understanding of spillover shocks across different market conditions (bearish, normal, and bullish). This approach aids investors in making informed decisions by providing insights into market dynamics under varying circumstances.

The remaining parts of the paper are structured as follows: Section 2 describes the data and initial analysis; Section 3 details the methodology employed in this study; Section 4 presents the empirical results, along with an analysis and discussion of the results; and finally, Section 5 wraps up the study.

## 2. Data and preliminary inquiry

Our research encompasses five smart transportation assets and five fossil-based energy assets to examine the dynamic and quantile connectivity between them (see Table 1 for details). To ensure uniformity across all sampled data series, we employ 2256 daily observations from 15 July 2013 to 20 June 2022. The beginning and the end dates of the samples, as well as the data frequency, are defined by the data availability. We employ logarithmic returns (∆In) throughout the estimations in this study to warrant the stability of the variable time series using theformula, $r_{t}=\ln\left(\frac{p_{t}}{p_{t-1}}\right),$ where $p_{t}$ is the daily closing price at time *t*.

**Table 1:** Variables’ definitions and data sources.

Dimensions	Variables’ name	Symbols	Data sources
Smart transportation assets	S&P Kensho Smart Transportation Index	SMTR	www.spglobal.com
	S&P Kensho Autonomous Vehicles Index,	AUVE	
	S&P Kensho Electric Vehicles Index	ELVE	
	S&P Kensho Advanced Transport Systems Index	ADTR	
	S&P Kensho Drones Index	DRON	
Fossil energy-relevant assets	S&P GSCI Equity Commodity Energy Index	COME	
	S&P GSCI Carbon Emission Allowances (EUA)	CEMA	
	Dow Jones Brookfield Electricity Transmission & Distribution Infrastructure Index	ELTR	
	S&P GSCI Petroleum	PTRL	
	Natural gas	NATG	www.investing.com

Note: GSCI stands for Goldman Sachs Commodity Index.

**Fig 2:**
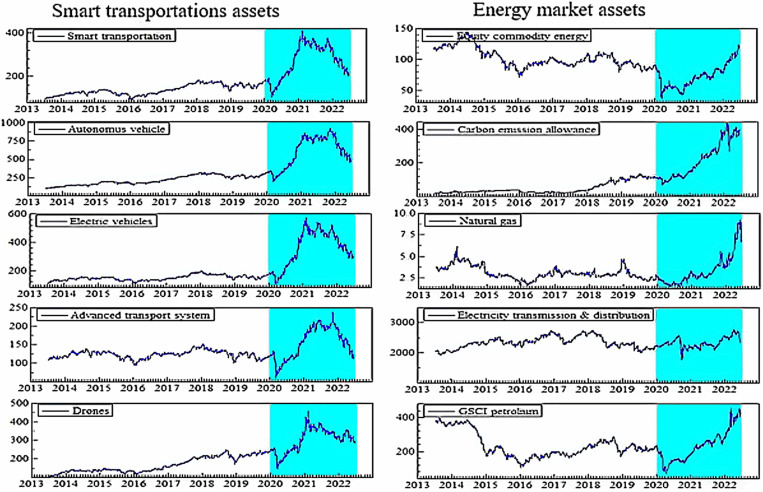
Plots of price dynamics. Note: The stock prices during stress such as COVID-19 (from 31 Dec 2019) and the Russia-Ukraine war (from 24 Feb 2022) are indicated by shaded areas.

Fig 2 illustrates the price trends of smart transportation and energy market assets. By observing the plots, we notice that all smart transportation stocks slowly grew till 2019. On the contrary, most fossil energy assets experience considerable ups and downs during the same periods. Both asset classes witnessed a slight drop in early 2020 due to COVID-19; following that, all smart transportation assets sharply increased up to 2021, followed by a decline beginning in early 2022 due to the Russia-Ukraine war. Most fossil energy assets, on the other hand, promptly rebounded from the second quarter of 2020 and continue to rise, with some volatility noticed in the early Russia-Ukraine war.

Table 2 presents the descriptive statistics, along with the results of stationarity and autocorrelation tests. Interestingly, all assets have positive mean returns, with AUVE and CEMA having the highest. However, the mean returns on the remaining assets are insignificant, though positive. NATG and ELTR represent the highest and lowest volatility, respectively. Except for NATG, all return series are negatively skewed, demonstrating the leptokurtic distributions with a heavier left-sided tail. The elevated kurtosis values across all return series confirm their non-normality, as verified by the Jarque-Bera test, which is significant at the 1% level. Thus, typical time-varying models may yield biased outputs when data series are non-normal with tail dependence, recommending a quantile-based approach.

**Table 2:** Summary statistics, stationarity, and autocorrelation tests.

	SMTR	AUVE	ELVE	ADTR	DRON	COME	CEMA	NATG	PTRL	ELTR
Mean	0.000	0.001	0.000	0.000	0.000	0.000	0.001	0.000	0.000	0.000
Maximum	0.110	0.101	0.107	0.116	0.088	0.151	0.161	0.400	0.174	0.093
Minimum	-0.147	-0.155	-0.129	-0.158	-0.133	-0.225	-0.194	-0.180	-0.333	-0.102
Std. Dev.	0.016	0.017	0.018	0.016	0.016	0.017	0.029	0.032	0.025	0.009
Kurtosis	11.401	10.031	8.876	13.659	12.779	25.379	8.058	16.179	27.862	22.498
Skewness	-0.794	-0.692	-0.374	-0.663	-0.617	-1.248	-0.598	0.873	-1.547	-0.568
Jarque-Bera	6868.978^*^	4825.954^*^	3297.840^*^	10841.740^*^	9129.430^*^	47644.840^*^	2538.155^*^	16607.600^*^	58975.600^*^	35841.870^*^
Q(10)	43.455^*^	36.795^*^	27.179^*^	50.646^*^	51.888^*^	39.618^*^	15.709^*^	9.821^***^	16.825^*^	61.303^*^
ADF	-15.385	-13.363^*^	-13.915^*^	-11.987^*^	-9.007^*^	-15.624^*^	-11.044^*^	-21.429^*^	-8.159^*^	-13.657^*^
PP	-47.954^*^	-48.124^*^	-47.077^*^	-47.420^*^	-48.527^*^	-49.644^*^	-50.595^*^	-49.934^*^	-49.063^*^	-46.681^*^

Notes: The table reports the outcomes of summary statistics, stationarity tests, and autocorrelation analysis for all returns series. The Augmented Dickey-Fuller and Phillips–Perron test statistics for unit root considering constant and trend at their level are symbolized by ADF and PP, respectively. The Ljung-Box (Q) test with 10 lags assesses series autocorrelation. Optimal lag lengths are determined using AIC. Std. Dev. refers to the standard deviation. ∆In refers to natural logarithm return. Significance levels of 1%, 5%, and 10% are indicated by

* ,

** , and

*** , respectively.

The Augmented Dickey-Fuller (ADF) and Phillips-Perron (PP) tests both reject the null hypothesis, indicating the stationarity of our return series. Additionally, the significant Ljung-Box (Q-10) statistics imply the autocorrelation problem, which ensures the non-linearity of our time series.

**Fig 3:**
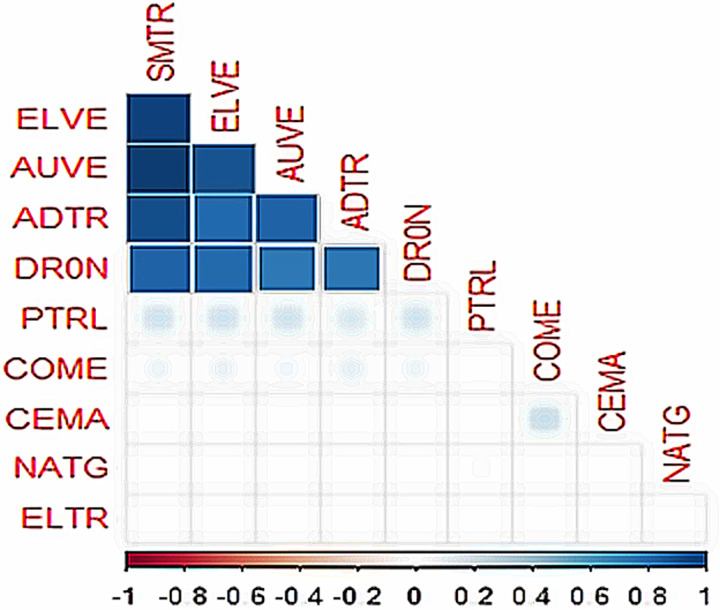
Visualization of Pearson’s correlation matrix.

Pearson’s correlation matrix, as shown in Fig 3, reveals a strong positive correlation between smart transportation assets. Fossil energy assets, such as PTRL and COME, have a weak positive correlation with smart transportation assets. However, the remaining transportation-energy market asset pairs have no correlation. These findings initially suggest that smart transportation assets may have hedge potential for fossil energy markets.

## 3. Methodology

### 3.1. Modeling of dynamic conditional correlation

In our study, we employ the dynamic conditional correlation (DCC) methodology pioneered by Engle [35] to analyze the time-varying conditional correlation between smart transportation and fossil energy assets. Contrasting to other multivariate GARCH models, DCC estimates the dimensionality by decomposing the conditional covariance matrix; hence, it is widely used [36,37]. The DCC technique is then integrated with the Glosten, Jagannathan, and Runkle [38] (GJR) method, a Generalized Autoregressive Conditional Heteroscedasticity (GARCH)-based approach. This hybrid method, known as DCC-GJR-GARCH, accommodates asymmetric effects such as leverage effects, where positive or negative shocks are associated with high or low volatility, respectively [36,39]. The following are the main procedures of DCC-GJR-GARCH, based on the Akaike Information Criterion (AIC):


$$r_{t}=\mu +\psi r_{t-1}+E_{t},E_{t}={\text{z}}_{t}h_{t},{\text{z}}_{t}\sim N\left(0,1\right),$$ (1)

where $r_{t}=\left[r_{i,t},\ldots ..,r_{n,t}\right]$ represents the (*n × 1*) vector of the studied assets’ returns. *μ* and *ψ* refer to the vectors of the constant terms and the coefficient of autoregressive terms, respectively. The vector for standard residuals is signified by $E_{t}=\left[E_{i,t},\ldots ..,E_{n,t}\right]$. Then, the conditional volatility, based on GJR-GARCH (1, 1) process, is designed to standardize the dynamics of variance as follows:


$$h_{i,t}^{2}=\text{ω }+\alpha E_{i-1}^{2}+\beta h_{i-1}^{2}+\Upsilon E_{i-1}^{2}I_{t-1},$$ (2)

where if $E_{t-1}<0$, then, $I_{t-1}=1$, otherwise $I_{t-1}=0$. The leverage term, represented by *Υ*, accounts for the asymmetrical effects of positive and negative events. When Υ>0, this specifies that the positive events affect less than the negative shocks. In Equation (2), ensuring the stability of the conditional volatility process can be achieved by setting the parameters: ω, α, β, and Υ if the criteria ω>0, α,β,Υ≥0, and $\Upsilon +\frac{\alpha +\beta }{2}<1$ are met.

The diagnostic Ljung-Box-tests (Qs-10) demonstrate that the GJR-GARCH specifications using student-t distribution are correctly specified because the residuals are free of autocorrelation issues. As a result, it is presumed that $E_{t-1}\left[E_{t}\right]=0$ and $E_{t-1}\left[E_{t},E_{t-1}^{'}\right]=H_{t}$, where $E\left[\cdot \right]=H_{t}$ embodies the conditional statement at time t. Therefore, for such a conditional variance-covariance matrix, nonetheless, $H_{t}$ can be represented as underneath:


$$H_{t}=D_{t}^{1/2}R_{t}D_{t}^{1/2},$$ (3)

where, $R_{t}$ denotes the time-varying correlation matrix (*n × n)*, and $D_{t}=diag\left(h_{i,t},\ldots .,h_{n,t}\right)$ specifies the diagonal conditional variance. Instead of $H_{t}$, the right-hand portion of Equation (4) is directly suggested by Engle [35] as a dynamic correlation structure:


$$R_{t}=diag{\left(X_{t}\right)}^{-1/2}X_{t}diag{\left(X_{t}\right)}^{-1/2},$$ (4)

$$X_{t}=\left(1-\alpha -\beta \right)K+\alpha \text{diag}{\left(X_{t-1}\right)}^{1/2}{\hat{\varepsilon }}_{i,t-1}{\hat{\varepsilon }}^{\text{'}}i,t-1diag{\left(X_{t-1}\right)}^{1/2}+\beta X_{t-1}$$ (5)

where the *n × n* unconditional covariance matrix for the standardized residuals ${\hat{\varepsilon }}^{'}i,t$ *is* *K,* and the method is called the DCC-GARCH model when α and *β* are the non-negative values considerable to α+β<1.

### 3.2. Quantile VAR specification

Given the non-normality and heavy tails observed in our data, which suggest the likely presence of tail dependence. Therefore, in this context, quantile connectedness approaches like quantile VAR (vector autoregression) are more suitable and preferable over traditional VAR models because they can account for asymmetric effects and extreme price movements, which are common in energy markets [40,41].

Unlike traditional VAR models, which primarily capture linear dependencies among variables, quantile VAR accommodates the heterogeneity and non-linear dynamics often exhibited in energy price data [40–43]. Additionally, compared to other VAR frameworks like TVP-VAR or LASSO-VAR, quantile VAR stands out for its ability to assess variable connectivity across a range of market conditions. By focusing on different quantiles, it provides deep insights into tail dependencies, offering a more nuanced understanding of how variables interact during extreme market conditions [40]. Moreover, quantile VAR delivers more accurate and detailed insights into connectedness because it is less influenced by outliers, making it particularly effective for analyzing time-domain connectedness in volatile markets [44]. However, we begin with quantile regression, following the methodologies of Koenker and Ng [45], Furno and Vistocco [46], and Jena et al. [47]. This approach allows us to study the dependency of the variable $\gamma_{t}$ on $x_{t}$ at every quantile (*τ*) of the conditional distribution of $\gamma_{t}/x_{t}$, as follows:


$$Q_{\tau }\left(\gamma_{t}|x_{t}\right)=x_{t}\beta \left(\tau \right),$$ (6)

where $Q_{\tau }$ refers to the *τ* ^th^ conditional quantile function of $\gamma_{t}$, where *τ* spans from 0 to 1 across quantiles. The explanatory variables are represented by $x_{t},\text{while}$ the relationship between $x_{t}$ and the *τ* ^th^ conditional quantile function of $\gamma_{t}$ is captured by $\beta \left(\tau \right)$. The parameter vector, $\beta \left(\tau \right),$ at the *τ* ^th^ conditional quartile $\left(\tau \right)$ is estimated using the subsequent expression:


$${\hat{\beta }}_{\left(\tau \right)}=argmin\overset{T}{\underset{t=1}{\sum }}(\tau -1_{\left\{y_{t}<x_{t}\beta \left(\tau \right)\right\}})\left|y_{t}<x_{t}\beta \left(\tau \right)\right|$$ (7)

then, the p^th^ order of the n-variable for the quantile VAR method is assessed as follows:


$$y_{t}=c\left(\tau \right)+\overset{p}{\underset{i=1}{\sum }}Bi(\tau )y_{t-1}+e_{t}\left(\tau \right),t=1,\ldots ,T$$ (8)

where, $\gamma_{t}$ is the n-vector of the dependent variable, while the n-vector of constants and residuals at quantile $\left(\tau \right)$ are reported by $c\left(\tau \right)$ and $e_{t}\left(\tau \right)$, respectively. $Bi\left(\tau \right)$ symbolizes the dependent variable’s matrix of lagged coefficients at quantile τ, where i=1,…,P. We then approximate ${\hat{\beta }}_{\left(\tau \right)}$ and $\hat{c}\left(\tau \right)$ by pretending the residuals, which adapt to the population quantile constraint, $Q_{\tau }\left(e_{t}\left(\tau \right)|y_{t-1},\ldots y_{t-p}\right)=0$. In Equation (9), we present the response *y* for the population of *τ* ^th^ conditional quantile. Furthermore, it enables us to estimate an equation-by-equation at each quantile τ afterward.


$$Q_{\tau }\left(\gamma_{t}|y_{t-1},\ldots \gamma_{t-p}\right)=c\left(\tau \right)+\overset{p}{\underset{i=1}{\sum }}Bi(\tau )y_{t-1}$$ (9)

#### 3.2.1. The connectedness estimates at each quantile.

In this sub-section, we compute different estimations of interconnectedness at every quantile $\left(\tau \right)$, following the groundbreaking work by Ando et al. [48], which updates the mean-based estimates developed by Diebold and Yilmaz [49]. We consider three return quantiles to capture three market conditions, including lower return quantiles (0.05|0.05), normal market quantiles (0.50|0.50), and upper return quantiles (0.95|0.95). The quantile connectedness approach is also employed by others [37,47,50]. However, we first modify Equation (8) as an indefinite order vector moving average (MA) progression for the connectivity measurements at each quantile:


$$y_{t}=µ\left(\tau \right)+\overset{\infty }{\underset{s=0}{\sum }}A_{s}(\tau )e_{t-s}\left(\tau \right),t=1,\ldots ,T$$ (10)

here,


$$µ\left(\tau \right)={\left({\text{I}}_{n}-B_{1}\left(\tau \right)-...-B_{p}\left(\tau \right)\right)}^{-1}c\left(\tau \right),{\text{A}}_{s}\left(\tau \right)= \{\begin{array}{c} 0,s<0:I_{n},s=0\\ B_{1}\left(\tau \right){\text{A}}_{s-1}\left(\tau \right)+\cdot \cdot \cdot +B_{p}\left(\tau \right){\text{A}}_{S-p}\left(\tau \right),s=0 \end{array}$$

where the sum of residuals $e_{t}\left(\tau \right)$ is signified by ${\text{y}}_{\text{t}}$.

Second, unlike Su [50], we adopt the techniques of Koop et al. [51] and Pesaran and Shin [52], which are resilient to variable reordering. For a forecast horizon H, the generalized forecast error variance decomposition (GFEVD) of a variable owing to shocks of distinct variables is:


$$\theta_{ij}^{g}\left(H\right)=\frac{\sigma_{jj}^{-1}\Sigma_{h=0}^{H-1}{\left(e_{i}^{'}{\text{A}}_{S}\Sigma e_{j}\right)}^{2}}{\Sigma_{h=0}^{H-1}\left(e_{i}^{'}{\text{A}}_{S}\Sigma e_{j}\right)}$$ (11)

where $\theta_{ij}^{g}\left(H\right)$ is the influence of the *j* ^th^ variable on the variance of the forecast error of the variable *i* ^th^ to horizon *H*. The error vector’s variance matrix in the equation is defined by Σ, and the j ^th^ diagonal component of the Σ matrix is denoted by $\sigma_{jj}$. $e_{i}$ is a vector worth 1 for the i ^th^ component and 0 otherwise.

Then, we follow the steps below to standardize each entry of the variance decomposition matrix:


$$\theta_{ij}^{\sim g}\left(H\right)=\frac{\theta_{ij}^{g}\left(H\right)}{\Sigma_{j=1}^{N}\theta_{ij}^{g}\left(H\right)}$$ (12)

Third, this study considers GFEVD, which can formulate four estimations of connectedness at every quantile. Using the following way, we denote the total spillover index (TSI) at quantile *τ*.


$$TSI\left(\tau \right)=\frac{\Sigma_{i=1}^{N}\Sigma_{j=1,i\neq j}^{N}\theta_{ji}^{\sim g}\left(\tau \right)}{\Sigma_{i=1}^{N}\Sigma_{j=1,}^{N}\theta_{ji}^{\sim g}\left(\tau \right)}\times 100$$ (13)

The total directional spillover index from index *i* to indices *j* at quantile 𝜏 is symbolized by “TO” as follows:


$$SI_{i\to j}\left(\tau \right)=\frac{\Sigma_{j=1,i\neq j}^{N}\theta_{ji}^{\sim g}\left(\tau \right)}{\Sigma_{j=1}^{N}\theta_{ji}^{\sim g}\left(\tau \right)}\times 100=TO$$ (14)

Conversely, to denote the total directional spillover index from indices *j* to index *i* at quantile 𝜏 “FROM” is used as follows:


$$SI_{i\leftarrow j}\left(\tau \right)=\frac{\Sigma_{j=1,i\neq j}^{N}\theta_{ji}^{\sim g}\left(\tau \right)}{\Sigma_{j=1}^{N}\theta_{ji}^{\sim g}\left(\tau \right)}\times 100=FROM$$ (15)

Then, at quantile *τ*, the net total directional spillover index (NSI) is as follows:


$$NSI_{I}\left(\tau \right)=SI_{i\to j}\left(\tau \right)-SI_{i\leftarrow j}\left(\tau \right)=NSI$$ (16)

Based on AIC criteria, the lag length criteria suggest one lag for the empirical investigation, and the forecast horizon is 10. We employ a 200-day rolling window estimator to evaluate the time-varying spillover of several return spillover metrics.

#### 3.2.2. Definitions of hedge and safe-haven assets.

To evaluate the hedging and safe-haven characteristics of smart transportation assets for energy assets, we follow the definitions set forth by Baur and McDermott [53]. In their framework, a hedge asset is characterized as either consistently having a negative correlation with another asset or portfolio (strong hedge) or uncorrelated on average (weak hedge). Similarly, a safe-haven asset is considered as one that is negatively correlated with another asset or portfolio only during specific periods, such as market downturns (strong safe-haven) or is uncorrelated during these periods (weak safe haven). These definitions have been applied in various recent studies [14,37,54,55].

## 4. Empirical findings and analysis

### 4.1. DCC-GJR-GARCH (1, 1) estimation

The outcomes of the DCC-GJR-GARCH (1, 1) model are exhibited in Table 3 (for detailed estimations, see Appendices 1–3). The table shows that the *⍺* (ARCH 1) and *β* (GARCH 1) parameters are significantly positive across all cases, with their sum being ≤ 1. The GJR (Gamma) term demonstrates a significant leverage effect for all selected stocks (except CEMA and NATG). The GJR-GARCH model, utilizing the student-*t* distribution, is well-fitted according to various diagnostic tests (e.g., Ljung-Box tests (Qs-10), standardized squared residuals, Li-McLeod, and multivariate Hosking) and information criteria presented in Panels B and C of the table.

**Table 3:** Results from DCC-GJR-GARCH (1, 1) estimation.

	Parameters	COME	CEMA	NATG	ELTR	PTRL
∆InSMTR	Avr. Corr.	0.760^*^	0.036	-0.002	-0.036	0.056
	Dcc (*a*)	0.019^*^	0.005	0.038^***^	0.000	0.016^**^
	Dcc (*b*)	0.979^*^	0.990^*^	0.329	0.451	0.978^*^
∆InAUVE	Avr. Corr.	0.685^*^	0.045^**^	0.009	-0.037^***^	0.077
	Dcc (*a*)	0.017^**^	0.009	0.009	0.000	0.016^**^
	Dcc (*b*)	0.0982^*^	0.526^*^	0.950^*^	0.607	0.977^*^
∆InELVE	Avr. Corr.	0.613^*^	0.032	-0.003	-0.037^***^	0.041
	Dcc (*a*)	0.023^**^	0.008^**^	0.012^***^	0.000	0.014^**^
	Dcc (*b*)	0.975^*^	0.980^*^	0.953^*^	0.006	0.980^*^
∆InADTR	Avr. Corr.	0.612^*^	0.044	0.002	-0.041^***^	0.029
	Dcc (*a*)	0.015^***^	0.007	0.000	0.032	0.011^**^
	Dcc (*b*)	0.985^*^	0.987^*^	0.912^*^	0.000	0.983^*^
∆InDRON	Avr. Corr.	0.732^*^	0.016	-0.008	-0.014	0.013
	Dcc (a)	0.017^*^	0.007	0.006	0.000	0.014^**^
	Dcc (b)	0.983^*^	0.985^*^	0.952^*^	0.885^*^	0.981^*^

Notes: Dcc (a) and Dcc (b) are the short-run and long-run DCC parameters, respectively. Significance levels of 1%, 5%, and 10% are indicated by

* ,

** , and

*** , respectively.

Overall, the DCC outcomes demonstrate that smart transportation proxies positively correlate with all fossil-based energy assets (excluding NATG and ELTR). SMTR correlates positively with COME, CEMA, and PTRL, with only COME showing a substantial positive dynamic connection. However, SMTR has a minor negative association with NATG and ELTR. Conversely, SMTR has a significant short- and long-term influence on COME and PTRL. The sum of short- and long-run persistence *(a+b)* is < 1, specifying the high volatility clustering between SMTR and COME and PTRL stock returns. The findings imply that SMTR might have short- and long-run spillover impacts on COME and PTRL. Furthermore, SMTR has a considerable short-run (long-run) impact on NATG (CEMA), resulting in a volatility spillover effect between them.

Consistently, AUVE and ADTR also significantly influence COME and PTRL in both the short- and long-run, demonstrating a high volatility clustering and spillover effect. Conversely, AUVE and ADTR exhibit long-run effects on CEMA and NATG at the 1% significance level. Interestingly, ELVE impacts all energy assets (excluding ELTR) in the short and long run. Hence, the shocks of the variables may spread between them. Finally, DRON has a substantial short- and long-run influence on COME and PTRL but only a long-run significant impact on CEMA, NATG, and ELTR at the 1% significance level.

**Fig 4:**
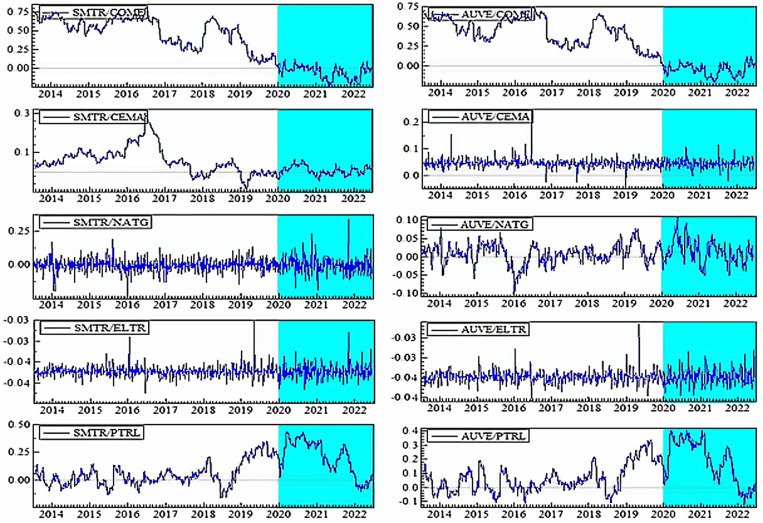
Plots of time-varying dynamic conditional correlations (SMTR, and AUVE vs. energy assets). Note: The shaded zones specify the turbulence period (COVID-19 and Russia-Ukraine conflict).

**Fig 5:**
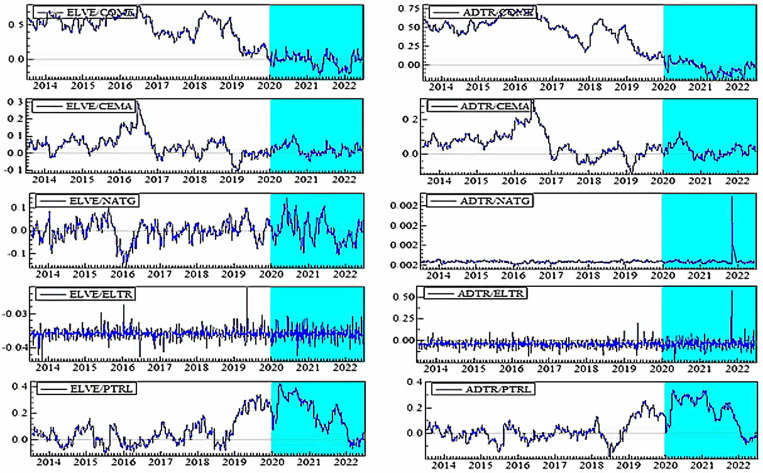
Plots of time-varying dynamic conditional correlations (ELVE, and ADTR vs. energy assets). Notes: See Table 4.

**Fig 6:**
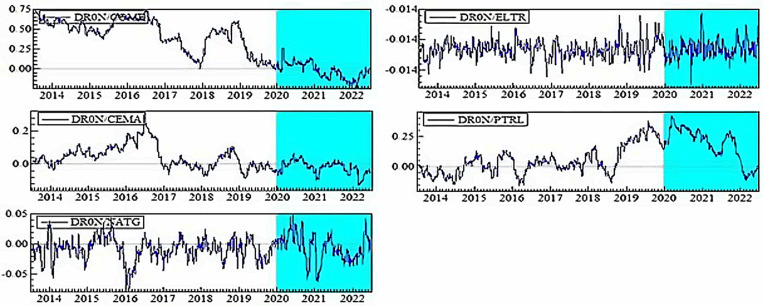
Plots of time-varying dynamic conditional correlations (DRON vs. energy assets). Notes: See Table 4.

Figs 4–6 plot the dynamic conditional correlations, suggesting a time-varying association between smart transportation and energy assets. Remarkably, the correlations suggest a structural shift for most smart transportation and energy pairs during crises, indicating that the link between the markets may become more susceptible during crisis periods. Fig 4 demonstrates that COME is substantially positively linked with SMTR and AUVE before turbulence, but the relationships turn negative during COVID-19 and remain until 2021. These findings imply that SMTR and AUVE do not provide a hedge for COME during normal periods but offer a modest safe-haven option during turbulences: COVID-19 and the Russia-Ukraine war. Similarly, SMTR does not offer a hedge for CEMA but provides a slight safe haven during the COVID-19 crisis. In contrast, AUVE offers no protection against CEMA because it is strongly associated with it throughout the study period. Likewise, both SMTR and AUVE fail to protect NATG since they have an average positive correlation with NATG over the study period.

On the contrary, both SMTR and AUVE exhibit an extremely negative correlation with ELTR throughout the period, indicating the hedge and safe-haven role of SMTR and AUVE against ELTR. However, PTRL displays distinct patterns of connection with SMTR and AUVE. More obviously, during COVID-19, the average negative correlations switch to a strong positive territory. This demonstrates that SMTR and AUVE cannot safeguard investors during a PTRL market downturn, albeit they can poorly hedge PTRL market volatility in normal times.

Fig 5 depicts that during normal times, ELVE and ADTR have a high positive connection with COME, with a modest negative correlation during the pandemic. As a result, ELVE and ADTR can provide safe havens for COME. They cannot, however, function as a hedge or safe haven for CEMA because they have a positive or zero correlation most of the time. Furthermore, the connection between NATG and ELVE alternates between negative and positive areas; however, in the case of the ADTR-NATG pair, the link is considerably positive. These findings show that ELVE can only hedge the NATG volatility slightly, but ADTR cannot. Conversely, even during crises, ELTR shows a strong negative association with both ELVE and ADTR, showing that ELVE and ADTR have great potential to provide hedging and safe-haven advantages. ELVE and ADTR, on the other hand, are poor hedges for PTRL.

Finally, Fig 6 shows that DRON eschews hedging capabilities but serves as a safe haven for both COME and CEMA during COVID-19. In contrast, DRON acts as a strong hedge and safe haven for ELTR, as there is a continual negative connection throughout the period. Nevertheless, DRON offers a weak hedge and a safe-haven opportunity for NATG, but it can only serve as a weak hedge to PTRL.

Overall, we notice that all smart transportation assets serve as a strong safe haven for COME and ELTR during turbulence while also serving as a strong hedge for ELTR during normal times. With a few exceptions, most smart transportation assets offer a weak hedge and safe haven for NATG and a weak hedge for PTRL. To attain net-zero emissions targets, several countries have set a target of fully electrifying the transportation industry by 2050 [11,12,56]. Non-governmental organizations are also concerned with reducing carbon emissions in the transportation industry through innovative solutions like promoting electric vehicles [56]. Governments have well-planned policies with well-structured governance to foster this smart transportation industry since they have a mandate and special attention to this sector in order to accomplish environmental goals. Furthermore, as previously discussed, smart transportation assets are considered green and green asset investors are ethical and large, do invest for the long term, and seldom move to unethical assets during a crisis. As a result, this industry is less subject to financial catastrophes. All of these factors may favor the smart transportation assets’ performance, allowing them to function as a hedge and safe haven for most fossil energy assets.

### 4.2. Results of quantile VAR estimations

Table 4 (Panel A) displays the estimated directional, pairwise, and total connectedness index (TCI) among the variables for the lower return quantile. TCI values (see bottom right corner) yield an average of 86.70%, indicating that the assets are inextricably linked under bearish market conditions. The “TO others” row suggests that ELVE transmits the highest spillover (95.51%) to other variables, followed by SMTR (95.35%). Conversely, the “FROM others” column exhibits that COME (88.96%) obtains the highest spillover from other variables in the system, followed by SMTR (87.66%). The lowest spillover spreaders and receivers to and from other variables in the system are CEMA (74.65%) and ELTR (84.52%), respectively. When compared to fossil energy assets, smart transportation assets are the leading spillover transmitters and receivers to and from others. In the case of net spillover connectivity, the “NET” row unveils that smart transportation assets are net spillover transmitters (positive), whereas fossil energy assets are net spillover receivers (negative).

**Table 4:** Static spillover connectedness.

	SMTR	AUVE	ELVE	ADTR	DR0N	COME	CEMA	NATG	ELTR	PTRL	FROM others
Panel A: for a lower return quantile (0.05|0.05)											
SMTR	12.34	11.76	11.62	11.61	10.93	8.96	7.82	7.89	8.17	8.89	87.66
AUVE	11.79	12.44	11.55	11.19	10.72	8.95	8.03	8.05	8.39	8.89	87.56
ELVE	11.66	11.39	12.61	11.21	10.81	9.14	7.76	8.07	8.39	8.95	87.39
ADTR	11.75	11.10	11.37	12.92	10.85	9.20	7.73	8.04	8.15	8.88	87.08
DR0N	11.43	11.02	11.38	11.32	12.75	9.16	7.86	7.91	8.51	8.64	87.25
COME	10.52	10.12	10.51	10.68	10.26	11.04	8.44	8.68	8.84	10.91	88.96
CEMA	9.67	9.90	9.78	10.00	9.57	8.95	14.03	9.08	9.43	9.59	85.97
NATG	9.40	9.48	9.64	9.75	9.35	9.04	9.01	15.27	9.58	9.48	84.73
ELTR	9.29	9.40	9.63	9.59	9.41	9.23	9.07	9.20	15.48	9.69	84.52
PTRL	9.84	9.73	10.02	9.96	9.68	9.13	8.93	9.04	9.54	14.10	85.90
**TO others**	95.35	93.92	95.51	95.30	91.58	81.78	74.65	75.96	79.02	83.94	867.01
NET	7.70	6.35	8.12	8.22	4.34	-7.19	-11.31	-8.77	-5.50	-1.96	TCI
											**86.70**
Panel B: for normal return quantile (0.5|0.5)											
SMTR	23.23	19.69	18.00	17.27	14.06	4.92	0.30	0.24	0.27	2.02	76.77
AUVE	22.14	26.30	17.68	14.07	12.37	4.52	0.39	0.32	0.25	1.95	73.70
ELVE	19.94	17.39	25.66	14.68	13.53	5.39	0.43	0.43	0.30	2.25	74.34
ADTR	20.20	14.71	15.47	27.37	13.38	5.36	0.38	0.40	0.42	2.30	72.63
DR0N	17.91	14.02	15.47	14.67	29.64	5.21	0.40	0.34	0.29	2.06	70.36
COME	11.35	9.24	10.41	10.46	10.15	26.54	0.53	0.64	0.42	20.24	73.46
CEMA	3.01	3.02	2.33	2.89	2.41	0.75	78.65	1.35	1.18	4.42	21.35
NATG	1.00	1.02	1.05	1.24	1.06	0.74	0.93	90.71	1.02	1.23	9.29
ELTR	0.82	0.73	0.78	1.32	0.85	0.57	1.20	1.01	91.64	1.08	8.36
PTRL	3.14	3.19	2.97	2.58	3.13	1.19	0.88	1.00	0.96	80.95	19.05
**TO others**	99.51	83.01	84.16	79.19	70.95	28.64	5.46	5.72	5.11	37.55	499.30
NET	22.74	9.31	9.82	6.56	0.59	-44.82	-15.89	-3.57	-3.25	18.51	TCI
											**49.93**
Panel C: for upper return quantile (0.95|0.95)											
SMTR	13.12	12.33	12.15	11.97	11.44	8.81	7.68	7.29	6.90	8.31	86.88
AUVE	12.52	13.32	11.99	11.21	11.10	8.70	7.93	7.58	7.11	8.54	86.68
ELVE	12.30	11.91	13.56	11.58	11.34	9.04	7.55	7.34	7.08	8.29	86.44
ADTR	11.99	11.19	11.55	13.50	11.08	8.95	8.03	7.70	7.43	8.57	86.50
DR0N	11.60	11.12	11.51	11.28	13.59	8.97	8.11	7.75	7.39	8.66	86.41
COME	10.42	10.15	10.53	10.59	10.52	11.72	8.58	8.23	7.98	11.28	88.28
CEMA	9.39	9.42	9.41	9.57	9.54	9.02	16.10	9.00	8.80	9.75	83.90
NATG	9.11	9.13	9.37	9.76	9.50	9.01	9.44	16.19	8.79	9.70	83.81
ELTR	9.03	8.96	9.36	9.34	9.51	8.91	9.57	9.26	16.81	9.25	83.19
PTRL	9.51	9.55	9.67	9.62	9.87	9.13	9.17	8.96	8.58	15.94	84.06
**TO others**	95.87	93.76	95.53	94.92	93.90	80.55	76.07	73.12	70.07	82.36	856.15
NET	8.99	7.07	9.10	8.42	7.49	-7.74	-7.83	-10.70	-13.12	-1.70	TCI
											**85.61**

Notes: The table displays the pairwise directional connectedness between smart transportation and fossil energy assets across different return quantiles (0.05, 0.5, and 0.95, representing lower, normal, and upper quantiles). The “TO others” row indicates the total directional connectedness to other assets, while the “FROM others” column shows the total directional connectedness received from other assets. NET and TCI refer to the net directional connectedness and total connectedness index, respectively.

In the case of normal market conditions (Table 4, Panel B), the TCI value (49.93%) unveils that the variables are weakly associated with each other. Explicitly, SMTR, followed by ELVE, is the largest transmitter and receiver of spillovers, as seen by the values “TO others” and “FROM others.” However, all smart transportation assets have positive NET values, while all fossil energy assets (except PTRL) have negative values, showing that the smart transportation and fossil energy markets are net spillover transmitters and receivers, respectively.

Finally, the TCI value for the upper return quantile (Table 4, Panel C) is 85.61%, indicating that the assets are highly connected under bullish market conditions. SMTR (95.87%) and COME (88.28%) are the greatest transmitters and recipients of spillover, according to the “TO others” row and “FROM others” column. The “NET” row depicts smart transportation as net spillover transmitters and fossil energy assets as net shock receivers.

**Fig 7:**
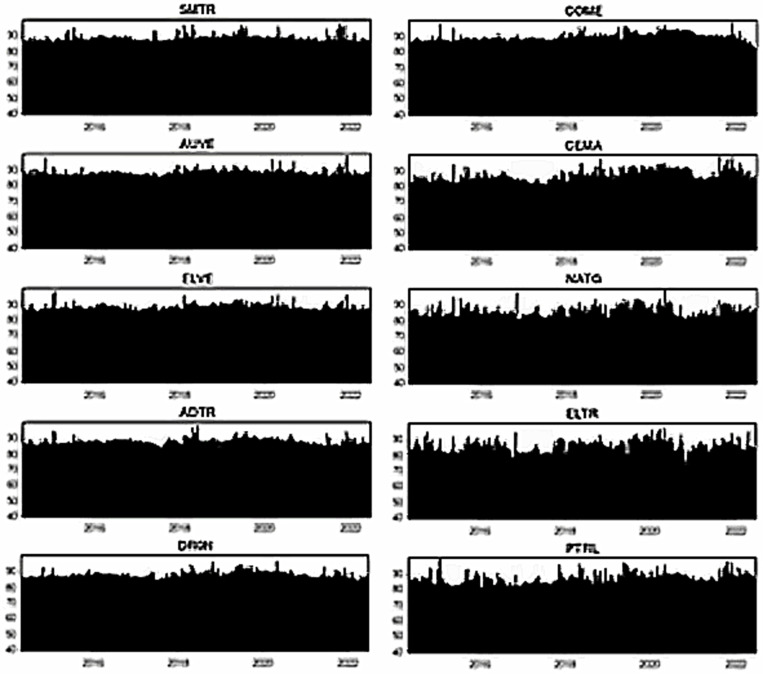
Return spillovers FROM others for the lower return quantiles (0.05|0.05).

**Fig 8:**
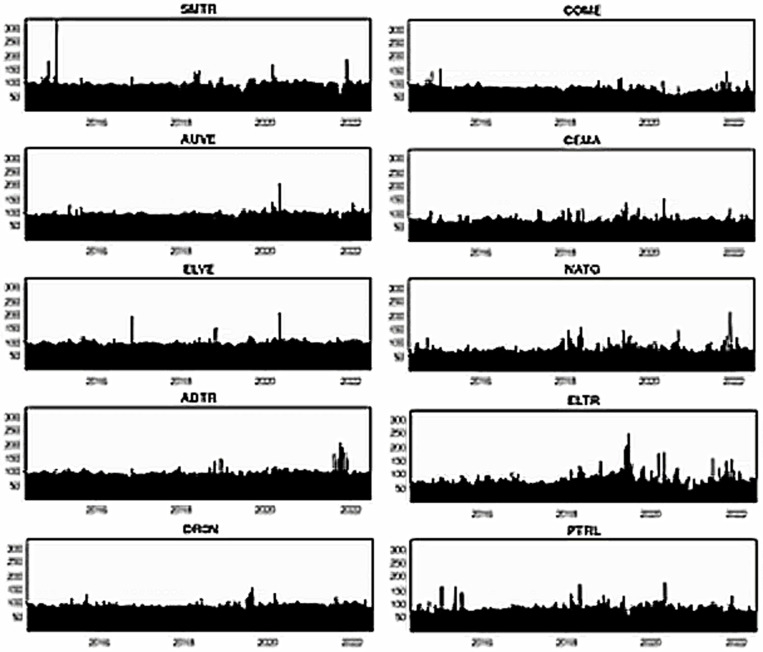
Return spillovers TO others for the lower return quantiles (0.05|0.05).

**Fig 9:**
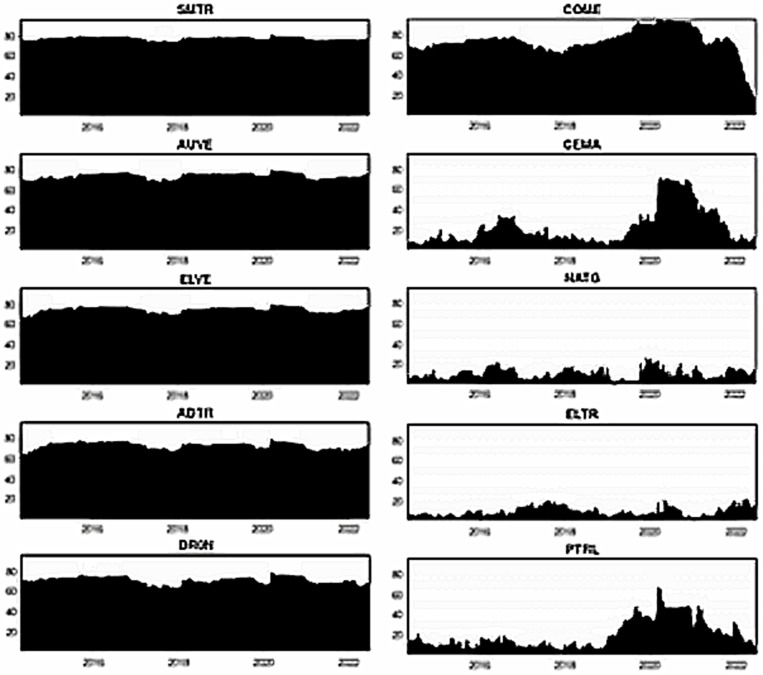
Return spillovers FROM others for the normal return quantiles (0.5|0.5).

**Fig 10:**
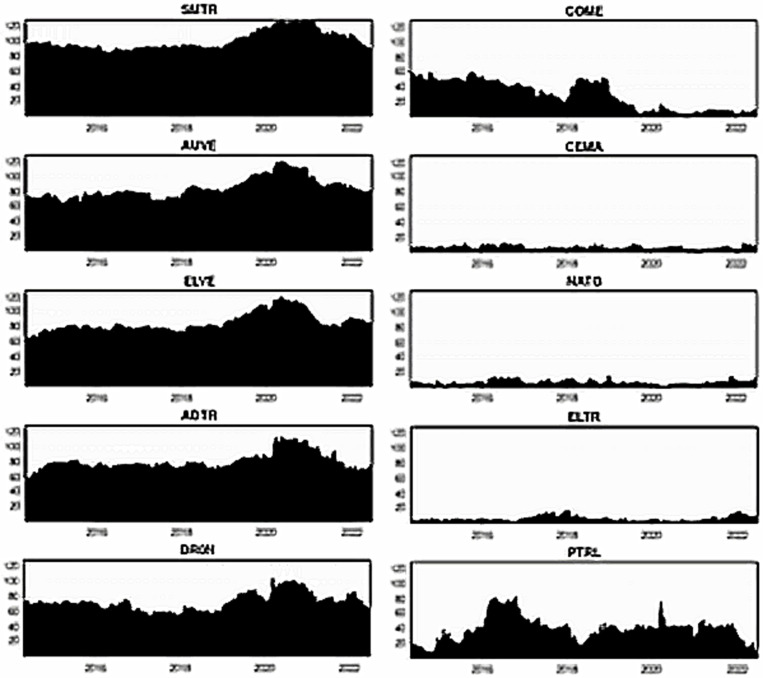
Return spillovers TO others for the normal return quantiles (0.5|0.5).

**Fig 11:**
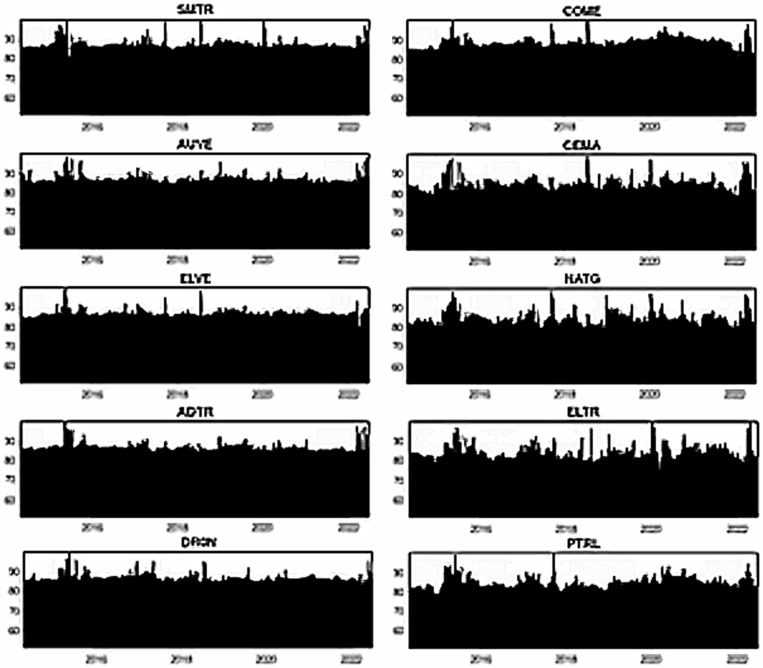
Return spillovers FROM others for the upper return quantiles (0.95|0.95).

**Fig 12:**
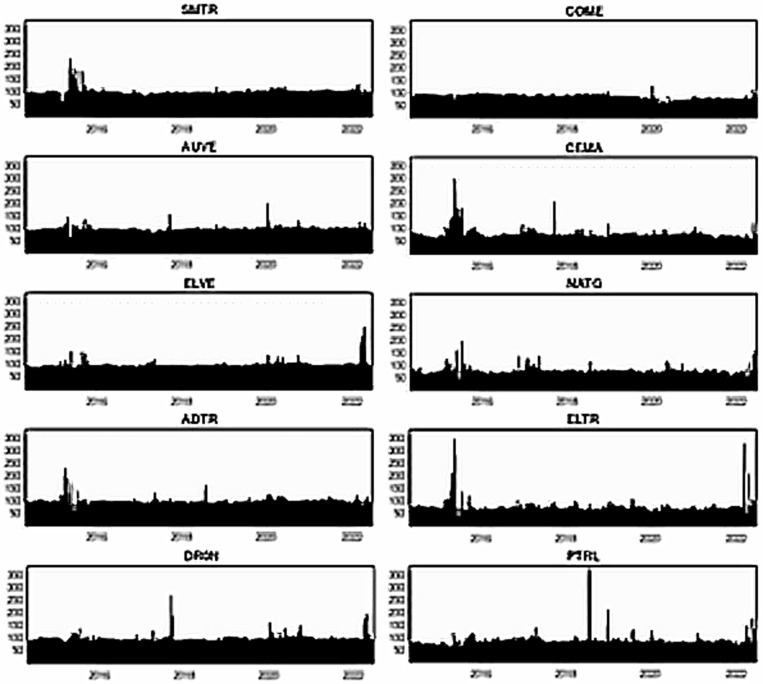
Return spillovers TO others for the upper return quantiles (0.95|0.95).

Figs 7–12 depict the dynamic directional spillover plots in three quantiles based on the “TO others” rows and “FROM others” columns in Table 4. Fig 7 shows that the directions of all assets’ spillovers do not alter considerably over time under the lower return quantile. Interestingly, all assets receive around 85% of the spillover from other factors during the study period. These findings indicate that during bearish market conditions, all assets experience significant spillover from other factors in the system. In contrast, the ‘TO others’ plots in Fig 8 show that all smart transportation assets transmit roughly 95% of spillover shocks, whereas fossil energy assets disseminate approximately 70% to 85% of return spillovers to others in lower return quantiles. These results demonstrate that during bearish market conditions, smart transportation assets are relatively higher spillover transmitters to other factors in the system.

In normal market conditions (depicted in Fig 9), smart transportation assets exhibit a similar pattern of spillovers, obtaining around 70% of spillovers from other factors. Conversely, fossil energy assets (excluding COME) show significant ups and downs in spillover over time, with relatively lesser spillover derived from others (less than 20%). However, COME, CEMA, and PTRL exhibit more spillover from others during 2019-early 2022. In contrast, in Fig 10, the ‘TO others’ plots reveal that smart transportation assets are the greatest spillover transmitters. However, the energy assets spread very low (less than 10%) to others (except for COME and PTRL, which spread around 30% up to 2019 and approximately 30% across the period, respectively). These findings suggest that in a regular market, smart transportation assets are the major transmitters and receivers of return spillovers.

Fig 11 shows that in bullish market conditions, all assets perform as substantial spillover recipients from others. Precisely, smart transportation and fossil energy assets receive about 95% and 80% of spillover shocks, respectively, from others in the system (see Fig 11), while transmitting around 95% and 75% of spillovers, respectively (see Fig 12). These findings imply that in bullish market situations, smart transportation assets are greater receivers and transmitters of return spillovers than fossil energy assets.

**Fig 13:**
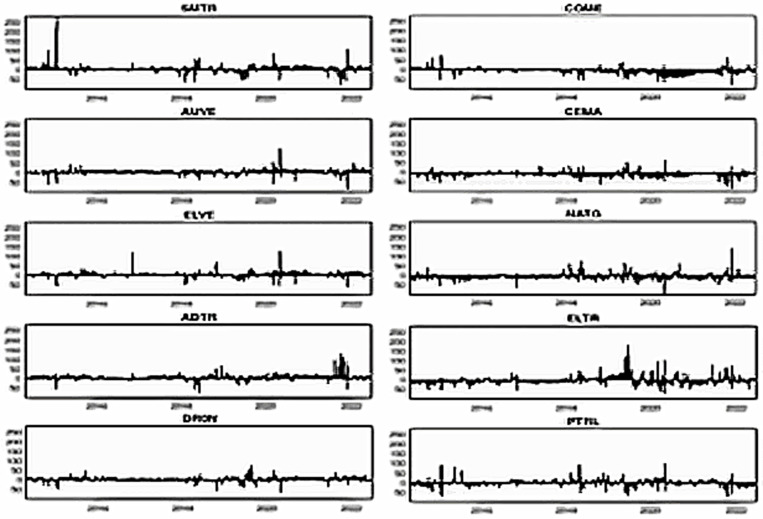
Net total directional return spillovers for the lower return quantiles (0.05|0.05).

**Fig 14:**
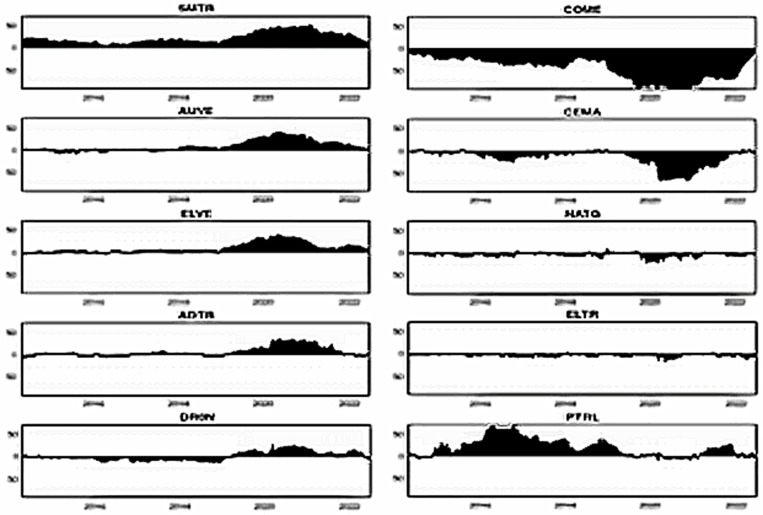
Net total directional return spillovers for the normal return quantiles (0.50|0.50).

**Fig 15:**
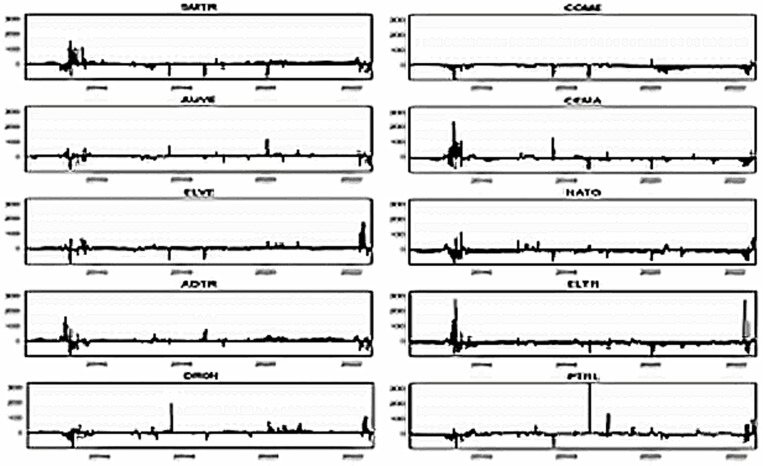
Net total directional return spillovers for the upper return quantiles (0.95|0.95).

Figs 13–15 portray the net total directional spillovers from/to other variables in the system over three quantiles (0.05, 0.50, and 0.95), respectively. Fig 13 shows that net total spillovers’ transmitting and receiving behaviors alter over time for lower return quantiles. Smart transportation assets have a positive net spillover on average (about 10%), whereas fossil energy assets have a negative net spillover on average (around 10%). During 2018–2021, however, fossil energy assets exhibit considerably larger negative spillover shocks. Therefore, in bearish market conditions, smart transportation assets are net transmitters of return spillovers, whereas fossil energy assets are net receivers.

On the contrary, Fig 14 for normal return quantile demonstrates that smart transportation assets, except DRON, have an average positive net spillover shock. In contrast, fossil assets, except PTRL, exhibit negative net spillovers. During 2020–2021, however, the net spillovers are significantly positive for smart transportation assets and negative for fossil energy assets (excluding PTRL). DRON has had a constant negative net spillover till 2019, but PTRL has had a positive net spillover throughout the same period. It is also observed that COME and CEMA exhibit significant negative spillovers, notably during the end of 2019-early 2022 period. Thus, in a normal market, smart transportation and PTRL assets are net transmitters of spillover shocks to others. However, fossil energy assets, except PTRL, are net recipients of spillovers from others.

Consistently, in Fig 15, we witness average positive and negative net total spillovers for smart transportation and fossil energy assets, respectively, in the bullish markets. Nevertheless, we see relatively higher net spillovers in 2015 and 2022 regarding both spreading and receiving for smart transportation and fossil energy assets. In addition, some minor non-continuous spillover adjustments are noticed throughout the period. These outcomes, therefore, reveal that smart transportation and fossil energy assets are the net transmitters and receivers of spillover shocks, respectively.

**Fig 16:**
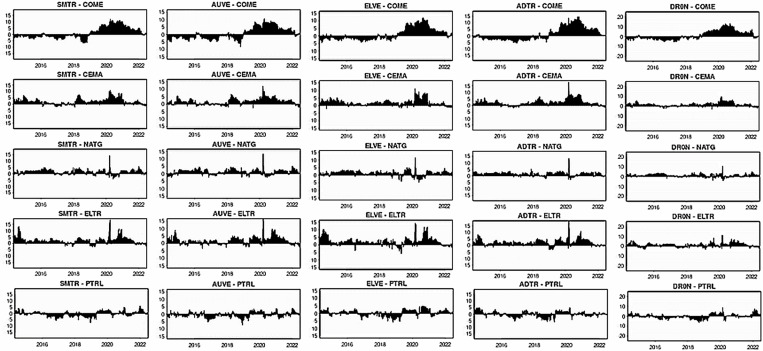
Net pairwise directional return spillovers for lower return quantile (0.05|0.05).

**Fig 17:**
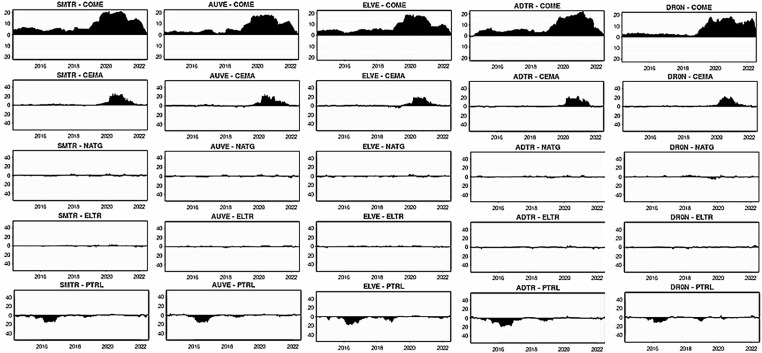
Net pairwise directional return spillovers for normal return quantile (0.5|0.5).

**Fig 18:**
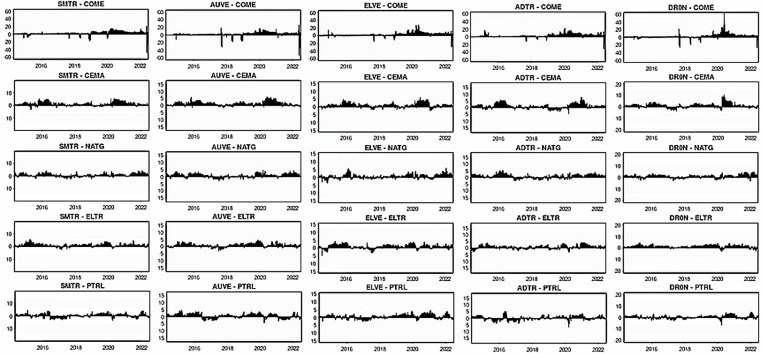
Net pairwise directional return spillovers for upper return quantile (0.95|0.95).

The dynamic net pairwise directional spillover connectivity under three quantiles are displayed in Figs 16-18, respectively. Fig 16 depicts the net pairwise spillovers in a bearish market. The findings show that during the normal period (up to 2018), all smart transportation assets are net receivers of spillover from COME but then become net transmitters of significant shocks to the COME when the global economy is in upheaval owing to COVID-19 and the Russia-Ukraine conflict. As a result, smart transportation assets are not attractive for portfolio managers in normal times but are appealing in times of turbulence for COME. Similarly, smart transportation assets are modest net shock transmitters for CEMA, NATG, and ELTR, with somewhat higher for CEMA and ELTR, especially during 2020, at roughly 7%, implying that smart transportation assets could be moderate diversifiers for these fossil energy assets. In the case of PTRL, the transmission and receipt of net shocks are inconsistent.

In Fig 17 for normal quantile, all smart transportation proxies are important net transmitters of shocks (about 20%) to COME, which grow over time, particularly between 2020–2021. Additionally, smart transportation assets significantly transmit net spillover to CEMA only during COVID-19. Smart transportation assets were the only receivers of net shocks from PTRL in 2016. In the case of NATG and ELTR, smart transportation assets have no significant role in sending and receiving net shocks across the period. These findings suggest that smart transportation assets may appeal to portfolio managers for hedging COME and CEMA, particularly during periods of turbulence.

In extreme higher quantile, Fig 18 depicts that smart transportation assets are net transmitters of shocks to fossil energy assets, albeit very low and inconsistent, indicating portfolio diversification opportunities. Nonetheless, in the case of COME and CEMA, smart assets are significant net spillover transmitters during 2020.

### 4.3. Pairwise network connectedness

**Fig 19:**
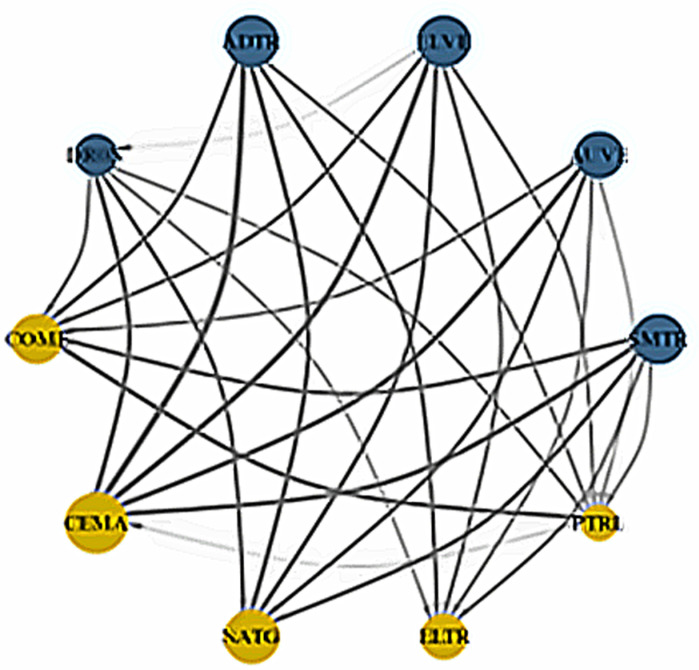
Network plots for lower return quantiles (0.05|0.05). Notes: The figure describes the pairwise connectivity among the variables across three quantiles. Blue nodes represent net transmitters of shockwaves, while yellow nodes indicate net receivers. Vertices are weighted by averaged net pairwise directional connectedness procedures. The node size reflects the weighted average net total directional connectedness. Arrows denote positive net directional connectedness, with the thickest arrows representing the strongest connections between variables.

**Fig 20:**
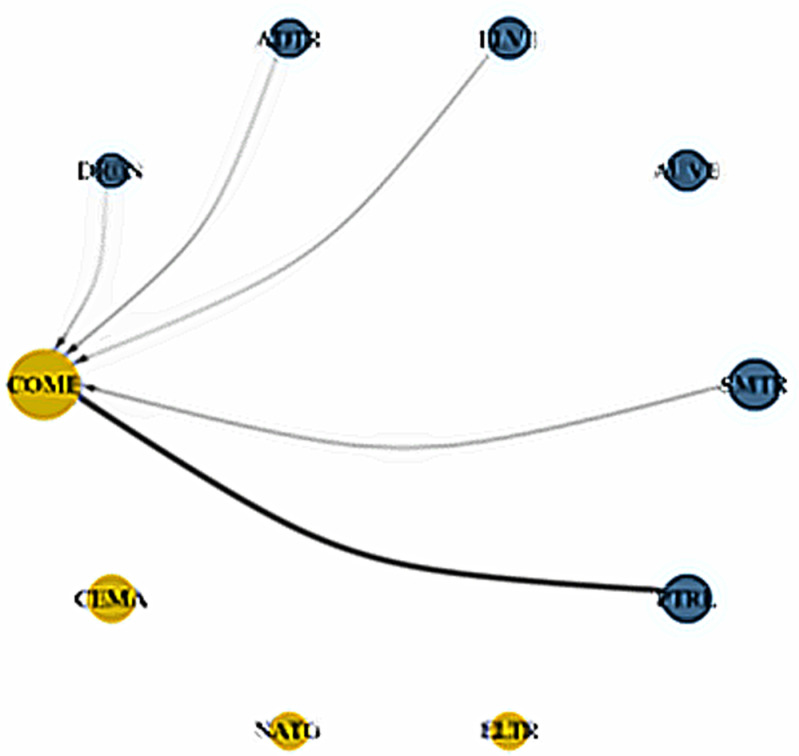
Network plots for normal return quantiles (0.50|0.50). Notes: For details descriptions of the network plot, please follow Fig 19.

**Fig 21:**
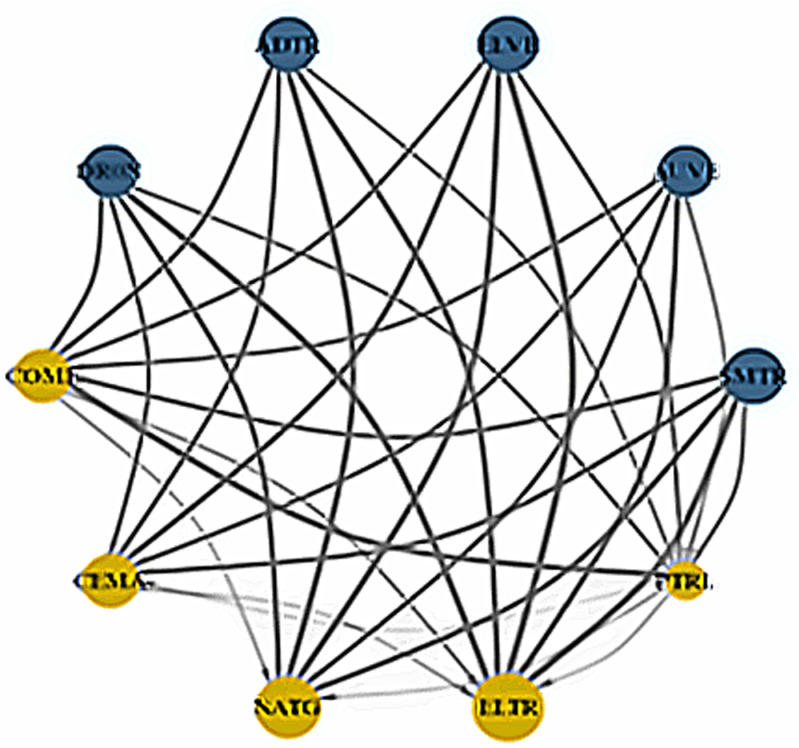
Network plots for upper return quantiles (0.95|0.95). Notes: For details descriptions of the network plot, please follow Fig 19..

In this section, we further investigate the network connectedness method of Diebold and Yilmaz [56] through the QVAR process to examine the pairwise spillover directional connectedness into three quantiles displayed, as reported in Fig 19, 20, and 21, respectively. The connectivity plots provide essential information about senders and recipients as well as the degree of connection under three different market scenarios. Fig 19 shows that in the lower return quantiles (0.05|0.05), smart transportation assets propagate shocks to fossil energy assets. Specifically, all smart transportation assets are important shock transmitters to CEMA, with ADTR and ELVE being the apex transmitters. Consistently, other energy assets, i.e., COME, NATG, ELTR, and PTRL, are also significant net spillover recipients from smart transportation assets. However, among the energy assets, only PTRL conveys shocks to COME and CEMA.

Conversely, in Fig 20, the normal market condition (0.5|0.5) reveals that COME is the sole recipient of spillovers from the smart transportation assets, excluding AUVE. Among all the energy assets, only PTRL transfers shocks to COME. Finally, the upper return quantiles (0.95|0.95) in Fig 21 show that all smart transportation assets are the net transmitters of shocks to energy assets. Nevertheless, PTRL functions as a shock transmitter toward energy assets, whereas COME is the thickest shock receiver. However, ELTR receives the least amount of shocks from COME and CEMA.

Overall, our analysis from the dynamic connectedness techniques and dynamic network plots highlights that smart transportation assets are significant transmitters of spillovers to fossil energy assets. Among fossil energy markets, the COME and CEMA markets emerge as the largest recipients of these spillover shocks, particularly from smart transportation assets and PTRL markets—even during the COVID-19 period. These findings suggest that smart transportation markets are more appealing to investors than fossil energy markets. As smart transportation assets are widely regarded as green investments, they are particularly attractive to climate-conscious investors. Furthermore, investor preferences for sustainable investments, such as those in the smart transportation sector, are evolving steadily [57]. Recognizing the severe impacts of climate change, investors are increasingly aware of the risks climate change poses to their portfolios. Green assets, such as those in the smart transportation sector, offer a dual advantage: they hedge against climate risks while delivering returns that are at least comparable to those of traditional investments [58]. On the other hand, as discussed earlier, fossil energy assets represent the inverse asset class to smart transportation assets, as policies supporting smart transportation often negatively impact the fossil energy industry [13]. As a result, smart transportation assets transmit significant spillover shocks to fossil energy markets.

Notably, during periods of market stress, these shocks are most pronounced in the COME and CEMA markets. The global emphasis on reducing greenhouse gas (GHG) emissions continues to drive the adoption of policies aimed at minimizing fossil fuel consumption and promoting green energy. For example, on September 23, 2019, UN Secretary-General António Guterres called on global leaders to enhance their climate commitments, targeting a 45% reduction in GHG emissions over the subsequent decade and achieving net-zero emissions by 2050 [59]. Similarly, on January 20, 2021, President Joe Biden issued Executive Order 13990, titled “Protecting Public Health and the Environment and Restoring Science to Tackle the Climate Crisis” [60]. These initiatives and others aim to support green energy sectors, including smart transportation while curbing reliance on fossil fuels. Consequently, such policy shifts may amplify the influence of smart transportation assets on fossil energy markets, particularly in the COME and CEMA sectors, as evidenced by the spillover shocks observed in our analysis.

## 5. Conclusions

This study uses daily time series data to explore the time-varying dynamic conditional correlation and quantile volatility connectedness between smart transportation and fossil energy markets. We first employ the DCC-GJR-GARCH (1,1) technique to study smart transportation’s hedge and safe-haven properties for fossil energy assets. Second, we use the quantile VAR approach to assess the spillover connectedness between the two asset classes.

Our DCC findings reveal a high volatility clustering and significant short- and long-run spillover effect between all smart transportations and COME and PTRL stock returns. However, with CEMA and NATG, smart transportation assets have only a long-term connectedness. Our findings further demonstrate that all smart transportation assets have robust safe-haven potentials for COME during crises—COVID-19 and the Russia-Ukraine conflict—while encompassing strong hedge and safe-haven benefits for ELTR. Nevertheless, smart transportation—SMTR, ELVE, and DRON—provide a poor hedge and safe haven for NATG, while SMTR, ADTR, and DRON present a weak hedge for PTRL. On the other hand, our QVAR analysis demonstrates that smart transportation assets are the primary transmitters of spillover shocks to fossil energy assets across all three quantiles, with COME and CEMA being the greatest receivers, even during turbulent times.

However, our findings have critical implications for both investors and policymakers. First, investors and portfolio managers, predominantly environmentally concerned, may diversify and safe-haven their portfolios by incorporating smart transportation stocks. Exclusively, our findings strongly advise investors to choose smart transportation assets as a strong hedge and safe-haven instrument for ELTR and the only safe-haven instrument for COME during crises. In addition, smart transportation investment can help to mitigate climate change. Second, our findings warn fossil energy investors of the potential volatility shocks posed by the smart transportation sector. Particularly, the high volatility clustering and both short- and long-run spillover effect between the sets will aid investors and portfolio managers in anticipating upcoming price or demand shocks, which is critical for counteracting suitable policy measures. Last but not least, our research demonstrates the investment potentiality of smart transportation sectors toward a smart world. However, the opposite may occur in the case of fossil energy demand for the transportation sector.

We acknowledge that this study has certain limitations. One notable limitation is the absence of hedge ratio analysis, which could have provided more actionable insights for investors seeking to manage spillover shocks between smart transportation and fossil energy assets. Additionally, the study focuses exclusively on a specific set of asset classes, leaving room to explore whether smart transportation assets can mitigate spillover shocks in other asset classes. Future research could address these limitations by incorporating hedge ratio and optimal portfolio weight analyses to offer more comprehensive insights into the hedging capabilities of smart transportation assets relative to fossil energy assets. Moreover, subsequent studies could expand on our findings by integrating additional datasets, exploring a broader range of variables, and employing more advanced methodologies, if any.

Given that this study offers the first evidence in this area, future researchers could validate and extend our conclusions by analyzing region-specific or country-specific smart transportation markets. Such studies could also explore their influence on energy markets and greenhouse gas emissions, further enriching the understanding of smart transportation’s role in sustainable finance and environmental impact.

## Supporting information

S1 Appendix Test results from DCC-GJR-GARCH (1, 1) estimation. Notes: Qs (10) are the Ljung–Box test statistics applied to the standardized residuals with 10 lags. Hosking and McLeod and Li multivariate Portmanteau statistics checks for the null hypothesis of no serial correlation (using 10 lags). The asterisks ‘*,’ ‘**,’ and ‘***’ indicate significance at 1%, 5%, and 10% levels, respectively.
(DOCX)

S2 Appendix Test results from DCC-GJR-GARCH (1, 1) estimation. Note: Please see the notes in Appendix 1.
(DOCX)

S3 Appendix Test results from DCC-GJR-GARCH (1, 1) estimation. Note: Please see the notes in Appendix 1.
(DOCX)

S1 Data Compressed Raw DATA. (XLSX)

S2 Data Data and code and estimation information. (DOCX)

S3 Data R-Codes-corr-plots. (TXT)

S4 Data DATA_Smart_Transportation_VS_Energy_assets. (XLSX)
