# Supplementary material for: Using smart transportation assets to hedge fossil energy markets: Evidence from quantile-based VAR approach
Source: PLoS One. 2025 May 9;20(5):e0317748. doi: 10.1371/journal.pone.0317748 (PMC12064208; doi:10.1371/journal.pone.0317748)
Supplement: S1 Appendix — Notes: Qs (10) are the Ljung–Box test statistics applied to the standardized residuals with 10 lags. Hosking and McLeod and Li multivariate Portmanteau statistics checks for the null hypothesis of no serial correlation (using 10 lags). The asterisks ‘*,’ ‘**,’ and ‘***’ indicate significance at 1%, 5%, and 10% levels, respectively. (DOCX) [file pone.0317748.s001.docx]

|  | SMTR vs. fossil energy assets | | | | | | | | | |  | AUVE vs. fossil energy assets | | | | | | | | | |
| --- | --- | --- | --- | --- | --- | --- | --- | --- | --- | --- | --- | --- | --- | --- | --- | --- | --- | --- | --- | --- | --- |
|  | SMTR | COME | SMTR | CEMA | SMTR | NATG | SMTR | ELTR | SMTR | PTRL |  | AUVE | COME | AUVE | CEMA | AUVE | NATG | AUVE | ELTR | AUVE | PTRL |
| Panel A: AR (1)-GARCH (1, 1) estimation | | | | | | | | | | | | | | | | | | | | | |
| Const. (M) | 0.000 | -0.000 | 0.000 | 0.002* | 0.000 | 0.001 | 0.000 | 0.000 | 0.000 | 0.000 |  | 0.000* | -0.000 | 0.000* | 0.002* | 0.000* | 0.001 | 0.000* | 0.000 | 0.000* | 0.000 |
| AR (1) | 0.008 | -0.028 | 0.008 | -0.067* | 0.008 | -0.0178 | 0.008 | 0.0709* | 0.008 | -0.026 |  | -0.001 | -0.028 | -0.001 | -0.067* | -0.001 | -0.0178 | -0.001 | 0.0709* | -0.001 | -0.026 |
| Const. (V) | 0.040** | 0.019* | 0.040** | **0.167***** | 0.040** | 0.105** | 0.040** | 4.237*** | 0.040** | 0.064** |  | 0.061** | 0.019* | 0.061** | 0.167*** | 0.061** | 0.105** | 0.061** | 4.237*** | 0.061** | 0.064** |
| ⍺ (ARCH 1) | 0.058* | 0.040* | 0.058* | 0.118* | 0.058* | 0.067* | 0.058* | 0.045*** | 0.058* | 0.063* |  | 0.055* | 0.040* | 0.055* | 0.118* | 0.055* | 0.067* | 0.055* | 0.045*** | 0.055* | 0.063* |
| β (GARCH 1) | 0.879* | 0.908* | 0.879* | 0.879* | 0.879* | 0.893* | 0.879* | 0.839* | 0.879* | 0.879* |  | 0.875*** | 0.908* | 0.875*** | 0.879* | 0.875*** | 0.893* | 0.875*** | 0.839* | 0.875*** | 0.879* |
| (⍺+ β) | 0.937 | 0.948 | 0.937 | 0.997 | 0.937 | 0.960 | 0.937 | 0.884 | 0.937 | 0.942 |  | 0.930 | 0.948 | 0.930 | 0.997 | 0.930 | 0.960 | 0.930 | 0.884 | 0.930 | 0.942 |
| GJR(Gamma) | 0.093** | 0.098* | 0.093** | -0.016 | 0.093** | 0.054 | 0.093** | 0.105** | 0.093** | 0.110* |  | 0.083** | 0.098* | 0.083** | -0.016 | 0.083** | 0.054 | 0.083** | 0.105** | 0.083** | 0.110* |
| Panel B: Diagnostic tests | | | | | | | | | | | | | | | | | | | | | |
| Qs (10) | 9.571 | 12.975 | 8.649 | 6.452 | 9.965 | 7.376 | 10.012 | 3.589 | 9.395 | 11.471 |  | 13.750 | 12.408 | 12.625 | 8.527 | 13.215 | 7.286 | 13.120 | 3.504 | 12.334 | 11.903 |
| Hosking (10) | 154.541*** | | 55.954** | | 41.813 | | 38.892 | | 95.467 | |  | 131.832*** | | 64.136* | | 53.024*** | | 39.730 | | 82.934 | |
| Li-McLeod(10) | 108.935 | | 55.947** | | 41.803 | | 38.118 | | 95.415 | |  | 131.781*** | | 64.097* | | 52.982*** | | 39.729 | | 82.885 | |
| Panel C: Information criteria | | | | | | | | | | | | | | | | | | | | | |
| Akaike | -11.821 | | -10.265 | | -10.144 | | -12.711 | | -10.984 | |  | -11.624 | | -10.134 | | -10.005 | | -12.571 | | -10.846 | |
| Shibata | -11.821 | | -10.266 | | -10.144 | | -12.711 | | -10.983 | |  | -11.624 | | -10.134 | | -10.005 | | -12.571 | | -10.846 | |
| Hannan-Quin | -11.806 | | -10.251 | | -10.129 | | -12.696 | | -10.968 | |  | -11.609 | | -10.119 | | -9.991 | | -12.556 | | -10.831 | |
