# Supplementary material for: Using smart transportation assets to hedge fossil energy markets: Evidence from quantile-based VAR approach
Source: PLoS One. 2025 May 9;20(5):e0317748. doi: 10.1371/journal.pone.0317748 (PMC12064208; doi:10.1371/journal.pone.0317748)
Supplement: S2 Appendix — Note: Please see the notes in Appendix 1. (DOCX) [file pone.0317748.s002.docx]

|  | ELVE vs. fossil energy assets | | | | | | | | | |  | ADTR vs. fossil energy assets | | | | | | | | | | | | | |
| --- | --- | --- | --- | --- | --- | --- | --- | --- | --- | --- | --- | --- | --- | --- | --- | --- | --- | --- | --- | --- | --- | --- | --- | --- | --- |
|  | ELVE | COME | ELVE | CEMA | ELVE | NATG | ELVE | ELTR | ELVE | PTRL |  | ADTR | COME | ADTR | | CEMA | ADTR | | NATG | ADTR | | ELTR | ADTR | | PTRL |
| Panel A: AR (1)-GARCH (1, 1) estimation | | | | | | | | | | | | | | | | | | | | | | | | | |
| Const. (M) | 0.000 | -0.000 | 0.000 | 0.002* | 0.000 | 0.001 | 0.000 | 0.000 | 0.000 | 0.000 |  | 0.000 | -0.000 | 0.000 | | 0.002* | 0.000 | | 0.001 | 0.000 | | 0.000 | 0.000 | | 0.000 |
| AR (1) | 0.025 | -0.028 | 0.025 | -0.067* | 0.025 | -0.0178 | 0.025 | 0.0709* | 0.025 | -0.026 |  | 0.026 | -0.028 | 0.026 | | -0.067* | 0.026 | | -0.0178 | 0.026 | | 0.0709* | 0.026 | | -0.026 |
| Const. (V) | 0.031** | 0.019* | 0.031** | **0.167***** | 0.031** | 0.105** | 0.031** | 4.237*** | 0.031** | 0.064** |  | 0.081* | 0.019* | 0.081* | | 0.167*** | 0.081* | | 0.105** | 0.081* | | 4.237*** | 0.081* | | 0.064** |
| ⍺ (ARCH 1) | 0.050* | 0.040* | 0.050* | 0.118* | 0.050* | 0.067* | 0.050* | 0.045*** | 0.050* | 0.063* |  | 0.103** | 0.040* | 0.103** | | 0.118* | 0.103** | | 0.067* | 0.103** | | 0.045*** | 0.103** | | 0.063* |
| β (GARCH 1) | 0.906* | 0.908* | 0.906* | 0.879* | 0.906* | 0.893* | 0.906* | 0.839* | 0.906* | 0.879* |  | 0.826* | 0.908* | 0.826* | | 0.879* | 0.826* | | 0.893* | 0.826* | | 0.839* | 0.826* | | 0.879* |
| (⍺+ β) | 0.956 | 0.948 | 0.956 | 0.997 | 0.956 | 0.960 | 0.956 | 0.884 | 0.956 | 0.942 |  | 0.929 | 0.948 | 0.929 | | 0.997 | 0.929 | | 0.960 | 0.929 | | 0.884 | 0.929 | | 0.942 |
| GJR(Gamma) | 0.069* | 0.098* | 0.069* | -0.016 | 0.069* | 0.054 | 0.069* | 0.105** | 0.069* | 0.110* |  | 0.083** | 0.098* | 0.083** | | -0.016 | 0.083** | | 0.054 | 0.083** | | 0.105** | 0.083** | | 0.110* |
| Panel B: Diagnostic tests | | | | | | | | | | | | | | | | | | | | | | | | | |
| Qs (10) | 9.137 | 11.944 | 7.174 | 8.563 | 7.723 | 7.286 | 7.547 | 3.649 | 7.153 | 11.218 |  | 5.097 | 13.347 | 5.190 | | 8.732 | 5.715 | | 7.400 | 5.882 | | 3.642 | .318 | | 11.473 |
| Hosking (10) | 144.806* | | 65.062 | | 33.643 | | 37.610** | | 84.068* | |  | 124.756* | | 43.716 | | | 30.605 | | | 24.310 | | | 81.623* | | |
| Li-McLeod(10) | 144.759* | | 65.025 | | 33.648 | | 33.560** | | 84.036* | |  | 124.728* | | 43.721 | | | 30.617 | | | 24.348 | | | 81.595* | | |
| Panel C: Information criteria | | | | | | | | | | | | | | | | | | | | | | | | | |
| Akaike | -11.545 | | -10.009 | | -9.887 | | -12.457 | | -10.719 | |  | -11.757 | | | -10.206 | | | -10.101 | | | -12.666 | | | -10.918 | |
| Shibata | -11.545 | | -10.010 | | -9.887 | | -12.457 | | -10.719 | |  | -11.757 | | | -10.206 | | | -10.101 | | | -12.667 | | | -10.918 | |
| Hannan-Quin | -11.530 | | -9.995 | | -9.872 | | -12.442 | | -10.704 | |  | -11.743 | | | -10.191 | | | -10.086 | | | -12.652 | | | -10.903 | |
