# Supplementary material for: Using smart transportation assets to hedge fossil energy markets: Evidence from quantile-based VAR approach
Source: PLoS One. 2025 May 9;20(5):e0317748. doi: 10.1371/journal.pone.0317748 (PMC12064208; doi:10.1371/journal.pone.0317748)
Supplement: S3 Appendix — Note: Please see the notes in Appendix 1. (DOCX) [file pone.0317748.s003.docx]

|  | DRON vs. Fossil energy assets | | | | | | | | | |
| --- | --- | --- | --- | --- | --- | --- | --- | --- | --- | --- |
|  | DRON | COME | DRON | CEMA | DRON | NATG | DRON | ELTR | DRON | PTRL |
| Panel A: AR (1)-GARCH (1, 1) estimation | | | | | | | | | | |
| Const. (M) | 0.000 | -0.000 | 0.000 | 0.002* | 0.000 | 0.001 | 0.000 | 0.000 | 0.000 | 0.000 |
| AR (1) | 0.019 | -0.028 | 0.019 | -0.067* | 0.019 | -0.0178 | 0.019 | 0.0709* | 0.019 | -0.026 |
| Const. (V) | 0.034* | 0.019* | 0.034* | 0.167*** | 0.034* | 0.105** | 0.034* | 4.237*** | 0.034* | 0.064** |
| ⍺ (ARCH 1) | 0.046* | 0.040* | 0.046* | 0.118* | 0.046* | 0.067* | 0.046* | 0.045*** | 0.046* | 0.063* |
| β (GARCH 1) | 0.895* | 0.908* | 0.895* | 0.879* | 0.895* | 0.893* | 0.895* | 0.839* | 0.895* | 0.879* |
| (⍺+ β) | 0.951 | 0.948 | 0.951 | 0.997 | 0.951 | 0.960 | 0.951 | 0.884 | 0.951 | 0.942 |
| GJR(Gamma) | 0.084* | 0.098* | 0.084* | -0.016 | 0.084* | 0.054 | 0.084* | 0.105** | 0.084* | 0.110* |
| Panel B: Diagnostic tests | | | | | | | | | | |
| Qs (10) | 4.890 | 10.748 | 5.721 | 9.057 | 5.663 | 7.450 | 5.790 | 3.551 | 5.835 | 11.569 |
| Hosking (10) | 153.293* | | 45.040 | | 42.928* | | 30.603 | | 83.618* | |
| Li-McLeod(10) | 153.245* | | 45.053 | | 42.920* | | 30.622 | | 83.589* | |
| Panel C: Information criteria | | | | | | | | | | |
| Akaike | -11.892 | | -10.395 | | -10.269 | | -12.847 | | -11.107 | |
| Shibata | -11.892 | | -10.395 | | -10.269 | | -12.848 | | -11.107 | |
| Hannan-Quin | -11.877 | | -10.380 | | -10.254 | | -12.833 | | -11.093 | |
