# Supplementary material for: Using smart transportation assets to hedge fossil energy markets: Evidence from quantile-based VAR approach
Source: PLoS One. 2025 May 9;20(5):e0317748. doi: 10.1371/journal.pone.0317748 (PMC12064208; doi:10.1371/journal.pone.0317748)
Supplement: S2 Data — (DOCX) [file pone.0317748.s005.docx]

**Manuscript Title:** **Using smart transportation assets to hedge fossil energy markets: Evidence from quantile-based VAR approach**

1. The data used are natural logarithm returns throughout the paper. The data file attached here is raw data.
2. For the visualization of Pearson’s correlation matrix, Figure 3, the R software is used. The code is attached named “Code for correlation plots in R”.
3. For modeling the Dynamic Conditional Correlation (DCC), OxMetrics7 is used.
4. We estimate the Quantile VAR (QVAR) and Pairwise network connectedness using David Gabauer’s online estimation platform. Please follow the link below: <https://sites.google.com/view/davidgabauer/econometric-code>
